# Correlations of Fat Content in Human Milk with Fat Droplet Size and Phospholipid Species

**DOI:** 10.3390/molecules26061596

**Published:** 2021-03-13

**Authors:** Beibei Duan, Eun-Sik Hong, Jung-Ah Shin, Yan Qin, Jeung-Hee Lee, Chi-Woo Lee, Ki-Teak Lee

**Affiliations:** 1 Department of Food Science and Technology, Chungnam National University, 99 Daehak-ro, Yuseong-gu, Daejeon 34134, Korea; just@cnu.ac.kr (B.D.); hes9730@o.cnu.ac.kr (E.-S.H.); sdfqy@cnu.ac.kr (Y.Q.); 2 Department of Food Processing and Distribution, Gangneung-Wonju National University, 7 Jukheon-gil, Gangneung, Gangwon-do 25457, Korea; jashin@gwnu.ac.kr; 3 Department of Food and Nutrition, Daegu University, 201 Daegudae-ro, Gyeonsan-si, Gyeongsangbukdo 38453, Korea; jeunghlee@daegu.ac.kr; 4 Maeil Innovation Center (MIC), Maeil Dairies Co., Ltd., 63 Jinwiseo-ro, Jinwi-myeon, Pyeongtaek-si, Gyeonggi-do 17714, Korea; beastory@maeil.com

**Keywords:** human milk fat, fat globule size, phospholipids, compositional correlation, HPLC-ELSD, ^31^P NMR

## Abstract

Fat globule size and phospholipid (PL) content in human milk (HM) were investigated. HM was classified into three groups depending on fat content (A < B < C). PL content (mg/100 g HM) was significantly higher in the C group (*p* < 0.05), indicating its positive relationship with HM fat content. When the PL content was normalized (mg/g fat), that of group A was significantly higher (*p* < 0.05) and fat droplet size in group C was slightly larger, suggesting that HM fat content is affected by fat droplet numbers to a larger extent than by fat droplet size. A correlation between PC and SM content in HM was observed regardless of fat content, while correlation between PE and either PC or SM increased in the order of C > B > A, hence the composition and content of PL species in HM varied according to its fat content.

## 1. Introduction

The fat in human milk (HM) is encapsulated by polar lipids and proteins, and exists in the form of fat droplets. Polar lipids are predominantly composed of phospholipids (PLs) and small amounts of other lipids, such as gangliosides, cholesterol, and ceramides [1]. Nowadays, increasing attention has been devoted to studying the PL content of HM owing to its importance in infant health and development [2,3].

PLs are phosphate-containing lipids found in milk fat globule membranes (MFGM) comprised of a triple phospholipid-protein layer. Phosphate groups can be esterified by various small molecules to form a variety of phosphoglycerides, such as phosphatidylcholine (PC), phosphatidylethanolamine (PE), phosphatidylserine (PS), and phosphatidylinositol (PI), which are the dominant species in HM along with sphingomyelin (SM), composed mainly of ceramide with a phosphorylated choline head group. Additionally, PLs can be converted to their lyso-form under the catalysis of phospholipases. The distribution of PLs on the fat globule membrane is not uniform. PE, PI, and PS are predominantly concentrated on the inner surface of the MFGM, whereas PC, SM, and glycolipids, including gangliosides and cerebrosides, generally gather in the MFGM bilayer [4]. Studies have shown that various PLs have important effects on the body; for example, PC and PE are beneficial for the treatment of liver disease and the reduction of cholesterol, respectively [5,6]. PS is closely related to the development of the central nervous system, and dietary supplementation with PS can improve cognitive abilities [7]. As an animal-specific PL, SM participates not only in the inflammatory response but also plays an important role in intestinal maturation of lactating infants [8,9]. Therefore, the study of the content and composition of PLs in HM is of great significance; in particular, such studies can provide guidance for the design of infant formula.

So far, PL concentrations in mature HM samples have been studied in various countries and regions [10,11,12,13,14], providing information on PE (5.2–9.9 mg/100 mL), PI (0.7–4.0 mg/100 mL), PS (0.8–4.3 mg/100 mL), PC (2.6–6.0 mg/100 mL), and SM (6.8–10.3 mg/100 mL). In addition, studies on the factors affecting PL concentration in HM, such as lactation stages, gestational ages, and sex of the infant, have also been conducted [10,11,12,13,15]. Among these factors, the lactation period appears to be the most significant. For example, from colostrum (4 ± 3 d) to mature milk (90 ± 3 d), the contents of PE, PI, PS, PC, and SM in full-term HM decrease by 3.5%, 28.6%, 34.1%, 24.6%, and 4.6%, respectively [10]. Moreover, even in mature HM (lactation stage: 16–240 d), the content of PLs such as PC, PS, and PI were also significantly different (*p* < 0.05) [12]. Maternal diet likely affects the PL content in mature HM because phosphorus (P), which cannot be synthesized by the human body, is essential for the synthesis of P-containing compounds such as PLs. Furthermore, different PL species have been shown to exhibit a preference for certain fatty acid types [16], thus PE generally esterifies polyunsaturated fatty acids, while saturated and monounsaturated fatty acids are esterified by SM.

In the mammary gland, the synthesis of milk fat is a dynamic system. Synthetic fat is encased by a triple phospholipid-protein layer and released into HM in the form of fat droplets. Therefore, an increase in the fat content of HM may be related to either the fat droplet size or amount, or both. The fat globule size affects digestion and absorption in breastfeeding infants. For these reasons, it is meaningful to explore the impact of HM fat content on PLs and fat globule size. Although studies on these relationships are rare, the research by Mizuno et al. [17] established that an increase in fat content was principally due to increases in the number of milk fat globules rather than the size of milk fat globules. In contrast, studies on cow’s milk suggest that increases in fat content do not increase the membrane material secreted by the mammary gland secretory cells, and that the fat droplets expand before being covered by the plasma membrane of the secretory apical membrane [18].

Until now, only a few studies have reported on the content and composition of PLs in HM from lactating mothers in Korea. Therefore, quantitative analyses of PL will play an important role in the design of infant formula, especially for the benefit of infants who have no opportunity to breastfeed. In this study, 34 HM samples were screened according to their fat content, and fat droplet size and PL contents were quantified to explore their relationship with fat content. Additionally, correlations between PL species were evaluated to explore the influence of fat content on the composition and content of PL species in HM.

## 2. Results

### 2.1. Fat Droplet Size Distribution and Diameter (d_32_, and d_43_)

**Figure 1:**
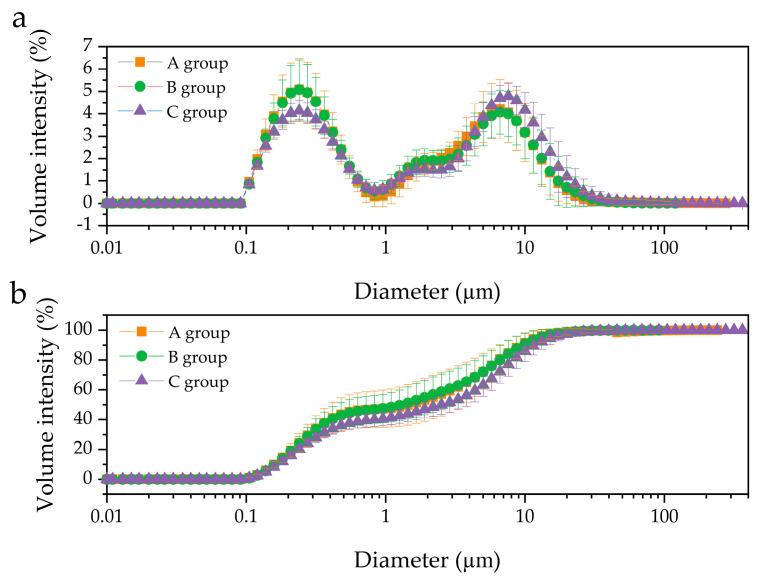
Comparison of the size distribution of human milk fat globules in different groups. (**a**) volume distribution, and (**b**) cumulative volume, distribution. A group, *n* = 11; B group, *n* = 11; C, group, *n* = 12.

Particle size distributions in three groups, A (0.11–239.88 μm), B (0.11–120.23 μm), and C (0.11–363.08 μm), classified by milk fat content, were determined by laser light scattering (Figure 1). All groups in this study showed bimodal size distribution of fat droplets, with diameters ranging from 0.1 to 1 μm and 1 to 91 μm. Additionally, the C group displayed a size distribution with a maximum volume (4.79%) at 7.59 μm, while the A and B groups showed maximum volume values (5.09% and 5.08%, respectively) at 0.24 μm.

**Figure 2:**
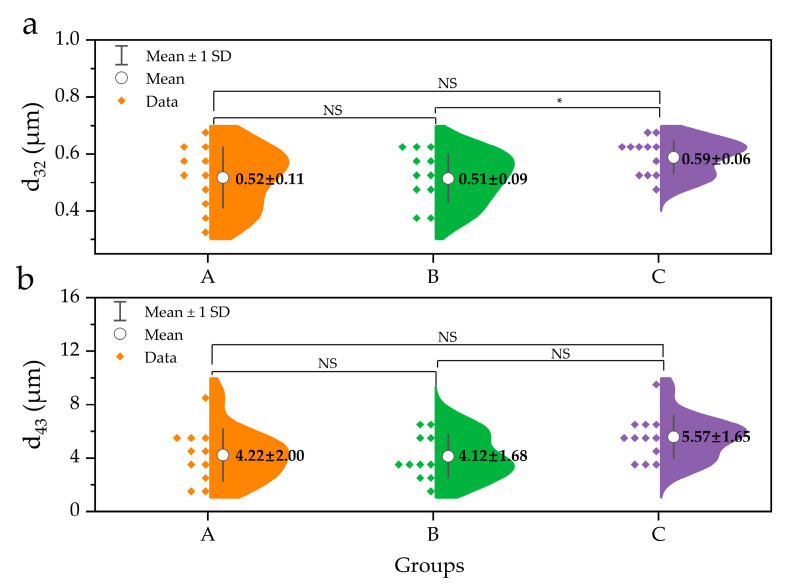
Size comparison of human milk fat globule in different groups. (**a**) d_32_, surface-weighted mean diameter; (**b**) d_43_, volume-weighted mean diameter. * represents a significance of difference at *p* < 0.05. NS represents non-significant difference (*p* > 0.05). A group, *n* = 11; B group, *n* = 11; C group, *n* = 12.

**Figure 3:**
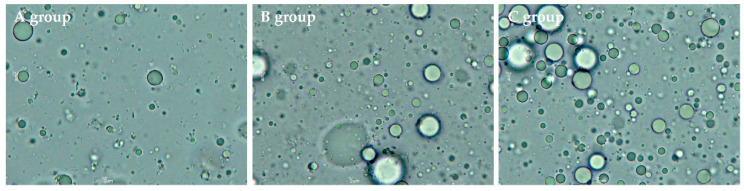
The optical microscopic images of human milk samples in different groups.

Furthermore, the volume-surface mean diameter (d_32_) and volume-weighted mean diameter (d_43_) between the three groups are illustrated in Figure 2. The d_43_, which is sensitive to larger particles, increased slightly from 4.22 μm in group A to 5.57 μm in group C (*p* > 0.05). Similarly, d_32_, which is sensitive to the presence of smaller particles, increased from 0.52 μm (in group A) to 0.59 μm (in group C) without significant differences between the 3 groups at *p* = 0.05 (Figure 2a), although d_32_ between group B and group C was significantly different (*p* < 0.05). In addition, the optical microscopic images of the samples are presented in Figure 3, wherein a larger number of fat droplets were observed in group C with a higher fat content than in the other groups.

### 2.2. Phospholipid Contents in Human Milk Samples

A quantitative HPLC analysis of PL content in mature Korean HM was performed using a power model for the quantitation curves according to a previous study [19], considering the nature of the ELSD response. In the case of PL content differences depending on the quantitation curve, linear and quadratic polynomial models were also used for comparison with the power model. However, the difference in the range of PL concentrations in the analyzed HM was negligible between the compared models. For example, average PC contents in HM were 2.28 mg/100 g and 2.14 mg/100 g from the linear and a quadratic polynomial models (data not shown), respectively, while 2.26 mg/100 g was obtained from the power model (Table 1). The total amount of PLs (i.e., sum of PE, PC, SM, and PS + LPC) in HM ranged from 4.89 to 17.85 mg/100 g, indicating that the PL concentration varied significantly between individual samples. The PE, PC, and SM contents ranged from 0.68 to 4.21 mg/100 g, 1.13 to 3.14 mg/100 g, and 2.35 to 8.32 mg/100 g, respectively (Table 1). In addition, SM was the most abundant PL, with a mean content of 5.11 mg/100 g HM, followed by PC (2.26 mg/100 g) and PE (2.00 mg/100 g).

**Figure 4:**
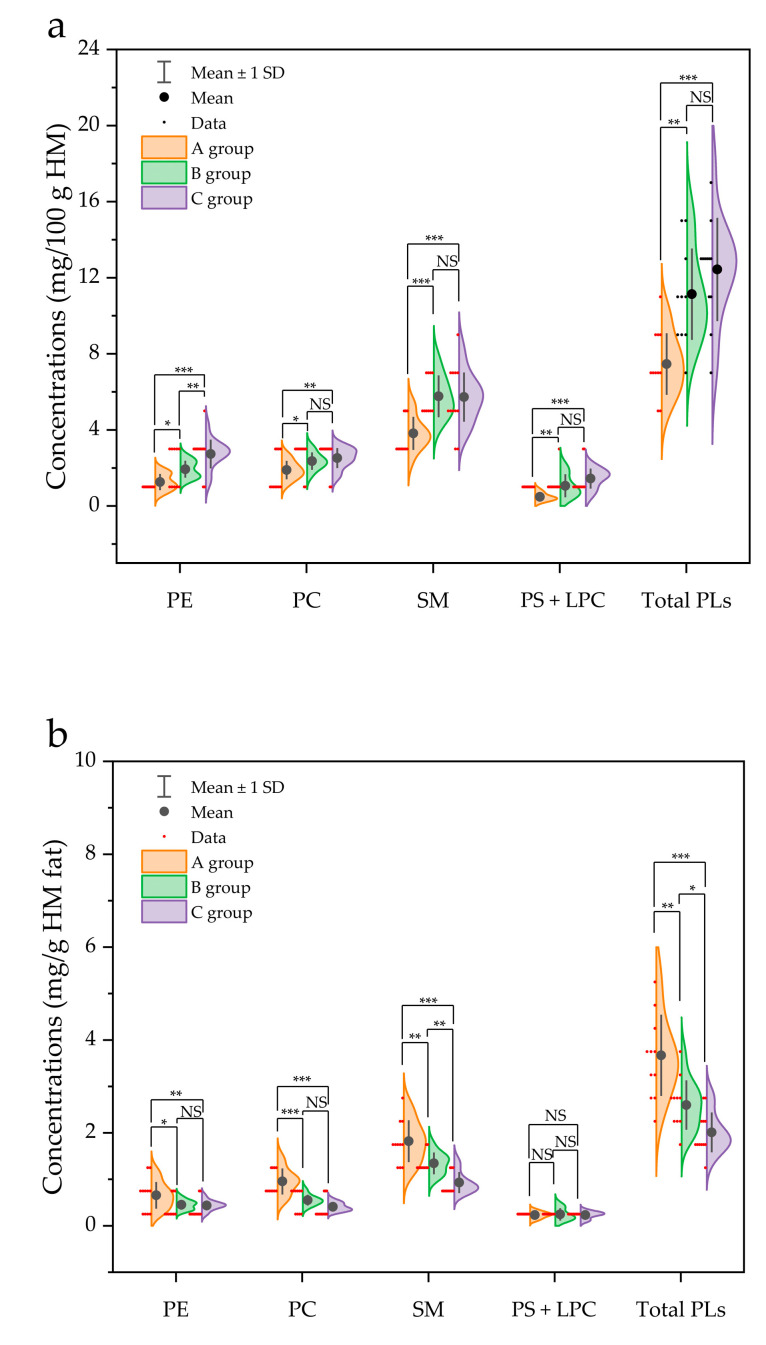
Comparison of the concentrations of phospholipid species in human milk. (**a**) Presented as mg per 100 g of human milk (HM), and (**b**) normalized for 1 g of HM fat in different groups (A group, *n* = 11; B group, *n* = 10; C group, *n* = 12. One sample in group B was omitted due to insufficient amount for analysis). *, **, and *** represent a significance of difference at *p* < 0.05, *p* < 0.01, and *p* < 0.001, respectively. NS represents non-significant difference (*p* > 0.05). PE, phosphatidylethanolamine; PC, phosphatidylcholine; SM, sphingomyelin; PS, phosphatidylserine; LPC, lysophosphatidylcholine; total PLs, sum of PE, PC, SM, PS and LPC.

**Figure 5:**
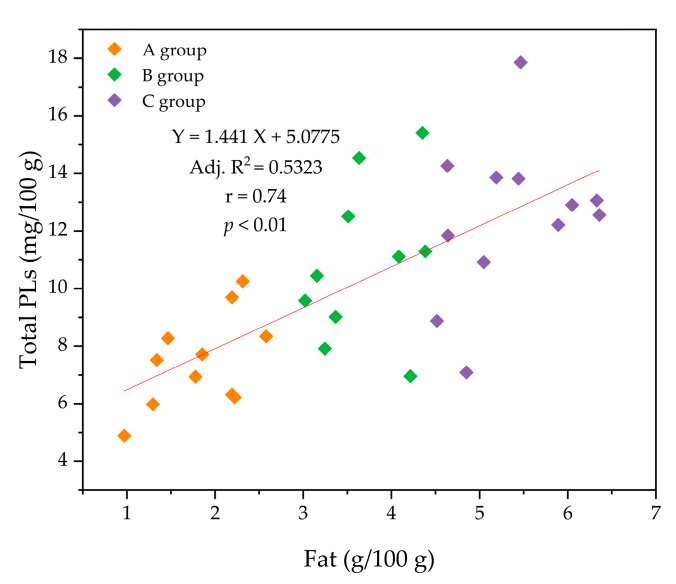
Correlation (*r*) between the concentrations of total phospholipids (PLs) and fat in human milk.

When comparing the total PL content (mg/100 g HM) between the three groups (A, B, and C), a significant increase was observed as the fat content of HM increased (*p* < 0.05). For example, the contents of PC (1.89 mg/100 g) and SM (3.82 mg/100 g) in group A were significantly lower than those in group C (2.52 and 5.73 mg/100 g, respectively) (Figure 4a). Additionally, a correlation between fat content and total PL content (*r* = 0.74; *p* < 0.01) was observed (Figure 5), indicating that a positive relationship exists between fat content and PL content in HM.

In addition, PL contents based on one g of fat (mg/g fat) are presented in Figure 4b. In contrast to the aforementioned result (PL content presented as mg per 100 g HM in Figure 4a), the total PL content normalized for 1 g of fat (mg/g fat) in group A was significantly higher than that in group C (*p* < 0.001) (Figure 4b). A similar trend was also observed for each individual PL, namely PE, PC, and SM. The content of PE (0.66 mg/g), PC (0.96 mg/g), and SM (1.82 mg/g) in A group was significantly higher than that in C group (0.44, 0.41, and 0.93 mg/g, respectively) (Figure 4b).

### 2.3. Correlations between Phospholipid Species

**Figure 6:**
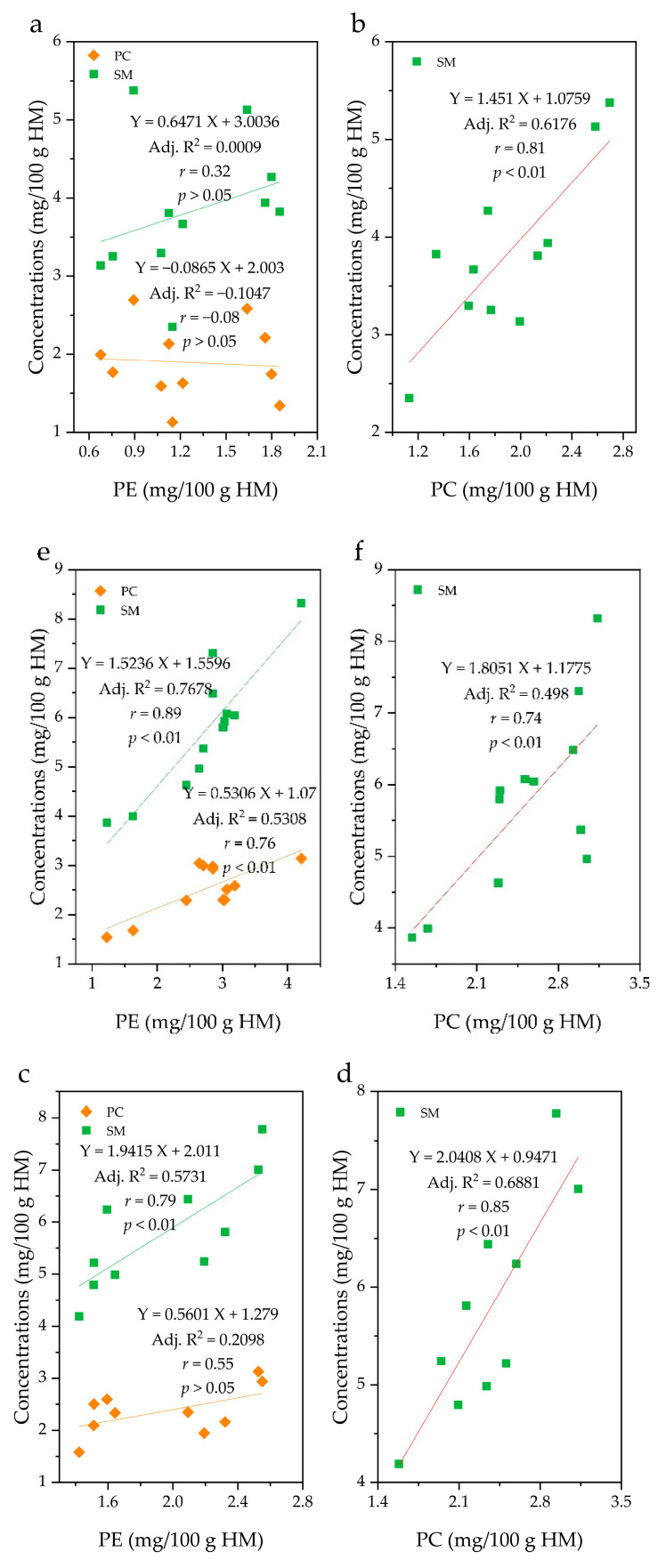
Correlations between the concentrations of phospholipid species in human milk (HM). A group = **a** and **b** (*n* = 11); B group = **c** and **d** (*n* = 10, one sample in group B was omitted due to insufficient amount for analysis); C group = **e** and **f** (*n* = 12). PE, phosphatidylethanolamine; PC, phosphatidylcholine; SM, sphingomyelin.

The correlation (*r*) between PC and SM content was high in all groups, with a range of 0.74–0.85. On the other hand, the correlation (*r*) between the PE and SM was in the order of C group (0.89) > B group (0.79) > A group (0.32), while that of PE and PC was in the order of C group (0.76) > B group (0.55) > A group (−0.08) (Figure 6). Interestingly, group A did not show a significant correlation between PE and PC or SM at *p* = 0.05, while the C group showed a significant correlation between PE and SM (*p* < 0.01), and between PE and PC (*p* < 0.01). In group B, a significant correlation was found between PE and SM (*p* < 0.01). In group C, the correlation between the PE and SM content (*r* = 0.89; *p* < 0.01) was as high as that between PC and SM (*r* = 0.74; *p* < 0.01). Because these groups have differing fat contents, it can be said that the differences in the correlation between PL species is dependent on the fat content.

### 2.4. Feasibility Study of ^31^P NMR for Phospholipids Quantification in Human Milk

Recently, ^31^P NMR has been used to determine the PL content in milk samples [10]. To explore the feasibility of ^31^P NMR for PL quantification in HM, PL contents were obtained from HPLC-ELSD and ^31^P NMR analysis and compared. In the HPLC-ESLD system, a YMC-Pack PVA-Sil column eluted with isopropanol, hexane, and water was used to separate PLs, which eluted in the order of PE, PC, SM, and PS + LPC (Figure S1a) [20]. To evaluate PL separation, a bare silica gel column instead of the YMC-Pack PVA-Sil column was tested under the same mobile conditions. However, peak resolution was not satisfactory during repeated injections, and the PL elution order differed from that of the YMC-Pack PVA-Sil column. YMC-Pack PVA-Sil column is bonded stationary phase, it can be washed with solvents of any polarity, from hexane through water, without altering the surface activity. PVA-Sil, which processes a polyvinyl alcohol (PVA) surface chemistry, exhibits better performance characteristics and selectivity in many cases. Usually, it can resolve compounds that behave poorly on silica. Considering that the YMC-Pack PVA-Sil column provided a higher resolution than the bare silica gel column [21], a YMC-Pack PVA-Sil column was used to separate the PLs in HM and infant formula in the present study.

In Figure S1, HPLC-ELSD chromatograms and ^31^P NMR spectra of fat from HM (Figure S1b,b’, respectively) and infant formula (Figure S1c,c’, respectively) are illustrated. For HM and infant formula, HPLC-ELSD could separate PE, PC, SM, and PS + LPC (Figure S1b,c). However, even though different species of PLs in infant formula (Figure S1c’) were identified for quantification by ^31^P NMR, PE and PS were barely detected (Figure S1b’) when HM was analyzed. Furthermore, when comparing the two methods for the quantification of PL content in HM and infant formula (Table S1), relative standard deviations (RSD) surpassing 20% occurred only for the PL content quantified in HM.

**Figure 7:**
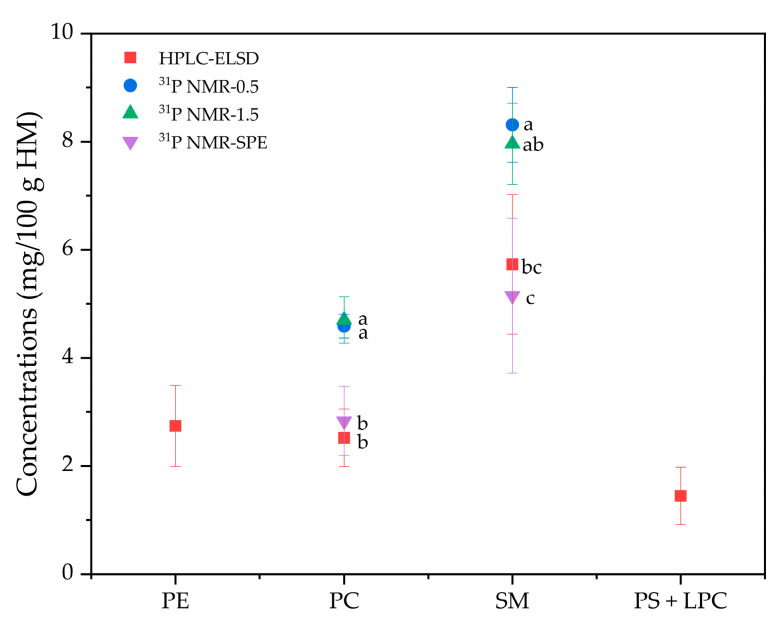
Comparison of the concentrations (mg/100 g HM) of phospholipid species. ^31^P NMR-0.5, ^31^P NMR analysis on 0.5 g of HM fat; ^31^P NMR-1.5, ^31^P NMR analysis on 1.5 g of HM fat; ^31^P NMR-SPE, ^31^P NMR analysis on HM fat applied to solid phase extraction (SPE); PE, phosphatidylethanolamine; PC, phosphatidylcholine; SM, sphingomyelin; PS, phosphatidylserine; LPC, lysophosphatidylcholine.

In contrast to infant formula, the analysis of PL content in HM did not provide satisfactory results, whereby ^31^P NMR indicated higher PC and SM content than observed with HPLC-ELSD, while PE and PS were not detected. Thus, the PL amounts acquired by ^31^P NMR were compared, whereby 0.5 g (^31^P NMR-0.5) and 1.5 g (^31^P NMR-1.5) of HM fat were placed in the NMR tube. HM fat was treated with SPE to increase the concentration of PLs before ^31^P NMR analysis (^31^P NMR-SPE). As shown in Figure 7, the concentrations of PC and SM inferred from ^31^P NMR-0.5 and ^31^P NMR-1.5 were higher than those from HPLC-ELSD, and minimal differences in PC and SM concentrations were noted despite the analyzed HM fat amount (i.e., 0.5 g and 1.5 g). Instead, PL concentrations in fat (% of fat) may be responsible for such differences. As shown in Figure 7, compared with ^31^P NMR-0.5 and ^31^P NMR-1.5, the results for PC and SM concentrations acquired from ^31^P NMR-SPE were comparable to those of HPLC-ELSD. For example, no significant difference in PC concentration was noted between ^31^P NMR-SPE and HPLC-ELSD analysis (*p* > 0.05).

## 3. Discussion

In this study, particle size distributions in different fat groups were explored, in which all groups showed bimodal size distribution of fat droplets, with diameters ranging from 0.1 to 1 μm and 1 to 91 μm (Figure 1). Previously, two primary distributions in mature HM were reported [15], while triple-particle distribution (at 0.1, 1, and 7 μm) has also been found [22,23], suggesting that the size distribution of fat droplets could differ among HM samples. Michalski et al. [23] observed the size distribution of HM fat globules by volume acquired during initial lactation days. Although HM was obtained from the same mother and early lactation HM was less stable than mature HM, the pattern of size distribution differed slightly depending on the collection time. Therefore, the origin of the analyzed HM (period, time, and number of collections) as well as personal differences (physiological and dietary variations) will affect the size distribution of fat droplets. Additionally, bile salt-stimulated lipase (BSSL), which is responsible for the degradation of milk fat in the neonatal intestine, exists naturally in HM [24]. Therefore, the difference in the size distribution of fat droplets could also arise from lipid hydrolysis by BSSL during HM preparation for analysis (e.g., vortexing or agitation).

Furthermore, although d_32_ and d_43_ increased slightly from group A to group C, no significant difference was observed (*p* > 0.05). The results depicted in Figure 1 and Figure 2 suggest that the size distribution and fat droplet diameter did not change significantly with variation in fat content, which is in contrast to those of previous studies [18,23], wherein an increase in fat content resulted in an increase in fat droplet size. On the other hand, it has been reported that an increase in fat content results chiefly from an increase in the number of milk fat globules rather than an increase in their size [17]. Fat in HM is surrounded by a triple phospholipid-protein layer and exists in the form of fat droplets in the HM environment. The most abundant PLs, such as SM and PC, are mainly gathered in the bilayer of the MFGM, while PE, PI, and PS are concentrated on the inner surface of MFGM [4]. The present findings indicate that an increase in fat content does not significantly affect the size of fat globules in HM. Nevertheless, the volume intensity of 0.1–0.6 μm fat droplets in the C group was smaller than that of the A and B groups, whereas droplets > 0.7 μm in size exhibited higher volumes in the C group than in the A and B groups. A similar tendency was observed at >6 μm (Figure 1a). Furthermore, the cumulative distribution by volume intensity of 0.1–182 μm fat droplets in group C was lower than that in groups A and B, while >182 μm fat droplets were only observed in group C (Figure 1b). In addition, although there was no statistically significant difference, d_32_ and d_43_ of group C were also larger than those of groups A and B (Figure 2). Meanwhile, there was no clear difference in d_32_ and d_43_ between the A and B groups (Figure 2); however, a slightly larger volume intensity was observed for the B group compared to that of the A group at >11.5 μm (Figure 1a). Similarly, the cumulative distribution by volume intensity of the 1–4 μm fat droplets in group B was slightly higher than that in group A (Figure 1b). Thus, if no difference is observed between the fat droplet size of HM with high fat content (group C) and that of HM with low fat content (group A), this is because the number of fat droplets in group C is greater than that in group A. Further, in order for a large number of fat droplets to exist, large amounts of PLs are needed to form emulsions such as HM. On the other hand, if even a small difference in fat droplet size is related to the fat content in HM, the C group with a high fat content would contain fat droplets of a larger size than group A, and therefore, the PL amount in the C group would also be greater than that in group A. The above assumptions are governed by a relationship between fat content and PL content in HM, necessitating further quantitative analysis of PLs in HM.

As for the contents of PLs in Korean HM, SM was the most abundant PL, with a mean content of 5.11 mg/100 g HM, followed by PC (2.26 mg/100 g) and PE (2.00 mg/100 g) (Table 1). The finding that SM was the most abundant PL in mature HM was in agreement with the literature [10,11,12,13,14,15,25,26,27], wherein a concentration range of 6.8–10.3 mg/100 mL was reported, accounting for 30.8%–43.3% of total PLs. However, in contrast to our results and those of previous studies [15,26,27], wherein PC content was found to be second to SM in HM, several studies [10,11,12,13,14,25] have reported that PE was second in abundance. Factors such as lactation stage, diet, and geographical differences as well as the consideration of lyso-PLs [10,11,12,13,26,27] may underlie these discrepancies. In addition, the content of PC (mean, 2.26 mg/100 g HM; ranges, 1.13–3.14 mg/100 g HM) in this study was comparable to that reported for HM from lactating mothers from Malaysia (mean, 2.6 mg/100 mL; ranges, 2.1–3.0 mg/100 mL) [11], but lower than that in HM from China (mean, 4.7 mg/100 mL; ranges, 4.4–5.2 mg/100 mL) [10], Spain (mean, 4.8 mg/100 mL; ranges, 3.6–6.6 mg/100 mL) [12], and Singapore (mean, 5.3–6.0 mg/100 mL; ranges, 3.2–9.6 mg/100 mL) [13,14]. The PE content in this study (mean, 2.00 mg/100 g HM; ranges, 0.68–4.21 mg/100 g HM) was lower than that reported in several other studies (3.1–11.8 mg/100 g or 100 mL HM) [10,11,13,14]. Nevertheless, the composition of PL classes (% of total PLs) in HM was comparable to that reported in the literature [10,11,12,13,14,15,26,27]. A homogenization procedure (agitation after thawing) is generally performed on frozen samples prior to further analysis, during which there is a possibility that PLs may be hydrolyzed to LPC by phospholipases naturally present in HM. In addition, along with the variability in the extraction and separation methods of PLs, other factors, such as maternal diet, lactation period, collecting time, and geographical origin are likely to also affect the PL concentrations in HM. Hence, a relatively wide range of PL concentrations would be expected in HM.

A significant increase in the contents of PLs (mg/100 g HM) was observed as the fat content of HM increased (*p* < 0.05) in this study. A similar result was reported by Zou et al. [15], wherein the fat content in HM increased with increasing polar lipid content. The higher PL content (mg/100 g HM) in group C than in group A was likely due to the higher milk fat content, suggesting that more lipid globules are released from the epithelial mammary glands. If such a phenomenon occurs, more amphiphilic lipids, i.e., PLs, are required to wrap each globule, incorporating them into lipid globules in HM. Similarly, as a component of fat globule membranes, a positive correlation between cholesterol content and fat content in the same HM samples was also found in our ongoing study. In HM, approximately 98% of milk fat is triacylglycerol (TAG), and a small portion (0.2−2%) is PLs that are located on the surface of the TAG core, along with specific proteins and cholesterol [28]. In the case of group C (the highest total fat content of HM), the total PL amount was high, but the normalized PL amount was low for the fat content, and the opposite trend was observed in group A (the lowest total fat content) (Figure 4). From the results, it can be concluded that the fat droplet size is relatively large in the HM of C group. Indeed, d_32_ and d_43_ in group C were larger than those in other groups, although statistical differences were not observed (*p* > 0.05), except for d_32_ between group B and group C. Moreover, previous studies have reported an inverse correlation between the diameter of milk fat globules and the total concentration of polar lipids [15,29]. However, previous studies have shown that the number of fat drops is related to the fat content of HM to a higher extent than to fat droplets size, wherein the milk fat globule size was not significantly different in foremilk (4.2 ± 1.0 μm) compared to that in hindmilk (4.6 ± 2.1 μm), although the fat content in hindmilk (8.6 ± 3.6%) was significantly higher than that in foremilk (3.7 ± 1.7%) [17]. Therefore, although no significant increase in the fat globule size was observed (*p* > 0.05), the fat droplet size as well as the number of fat droplets and individual factors, such as the TAG biosynthesis capability of the mammary gland and diet, all have some influence on the increase in the fat content of HM.

In addition, the correlations between PL species in different fat groups were explored, and high correlation between PC and SM content in HM was observed regardless of fat content, while the correlation between PE and either PC or SM increased in the order of C > B > A (Figure 6). SM can be classified as a phospholipid because it consists of a ceramide unit mainly with a phosphorylcholine moiety. Ceramide, which is synthesized in the endoplasmic reticulum, is composed of sphingosine and a fatty acid. Even though sphingosyl-phosphoryl ethanolamine containing an ethanolamine rather than a choline head group is analogous to SM, it is known that the level of SM produced by the phosphorylcholine polar head group attached to the ceramide backbone is 300−1500-fold higher than that produced by the ethanolamine head group in mammals [30]. Thus, the majority of SM in HM is believed to be ceramides containing phosphocholine groups.

Such a high correlation (*r*) between PC and SM may be related to the PL biosynthetic pathway. The Kennedy pathway suggested that SM can be formed directly by exchanging the head group of PC with ceramide, while PE can only be converted into PC through continuous methylation via a minor route [31]. Because PC contributes to the synthesis of SM as a choline donor [32], SM is related to PC to a higher degree than PE when considering the pathway, and such a biosynthetic pathway may explain why the correlation between the PC and SM content was higher than that between PE and SM in groups A and B. However, in group C, the correlation between the PE and SM content (*r* = 0.89; *p* < 0.01) was as high as that between PC and SM (*r* = 0.74; *p* < 0.01) (Figure 6e,f). Choline can be obtained from the diet and via de novo biosynthesis accomplished by continuous methylation of PE via a minor route [31]. Therefore, although the human body can endogenously produce PC containing a choline moiety from PE, predominantly in the liver, the amount is insufficient to meet the requirements of the human body [33], suggesting that humans need to intake choline from the diet. For example, animal foods, such as beef, milk, and eggs are rich in choline [34]. In addition, ethanolamine as the head group of PE and other minor PLs cannot be synthesized by mammals. Thus, the need for dietary intake of choline and ethanolamine suggests that the PL content in HM is affected by the maternal diet. The HM of mothers in the C group exhibited higher milk fat content than those in groups A and B, suggesting that differing genetic characteristics may exist, but it can be said that they could get sufficient nutritional supply because of the positive correlation between diet and fat content in HM [35,36]. If so, sufficient amounts of ingested ethanolamine are exhaustively utilized to synthesize PE in group C, likely resulting in an increase in the correlation between PC and PE content when compared with that in groups A and B.

Comparing the two methods (HPLC-ELSD and ^31^P NMR) for the quantification of PL content in HM and infant formula (Table S1), relative standard deviations (RSD) surpassing 20% occurred only for the PL content quantified in HM. One of the causes for this discrepancy was thought to be the level of PL in the samples. MacKenzie et al. [37] measured the PL content in dairy products by ^31^P NMR and found that ^31^P NMR was suitable for samples containing high levels of PLs, but that the measurement results may not be reliable for samples containing less than 1% PL. Relatively poor sensitivity of ^31^P NMR spectroscopy has also been reported by Helmerich et al. [38], who established that ^31^P NMR has a higher detection PL limit (1.4 mg/mL) than HPLC (0.02 mg/mL). As shown in Table S1, the total PL content in infant formula (2.47%) was more than 10 times that of HM (0.19%). Correspondingly, high PL levels in infant formula resulted in minimal differences in the results (mg/100 g) obtained from the two methods (Table S1).

In addition, ^31^P NMR analysis of PLs may be affected by analysis conditions [39], among which, variations in the amount of internal standard (IS) was studied. When 140 and 730 ppm of TPP (IS) were tested, TPP concentration had no significant effect on the determination of PLs in infant formula by ^31^P NMR (*p* > 0.05) in this study (data not shown).

In a previous study, Lanier et al. [40] used soy lecithin to evaluate ^31^P NMR accuracy, and found that the lower the PL level (% of fat), the lower the accuracy. In this study, the total PL obtained from HPLC-ELSD accounted for only 0.19% (% of fat) (Table S1), even though the sum of PE, PC, SM, and PS represents more than 80% of PLs in HM. Based on the present results, it can be speculated that PL content obtained by direct measurement of HM fat by ^31^P NMR may overestimate the actual content of PLs in HM. Alternatively, prior to ^31^P NMR analysis, SPE or acetone precipitation techniques should be practiced to increase the PL levels. Through these treatments, the feasibility of using ^31^P NMR to quantitatively analyze the contents of PLs in HM will be improved.

In addition, it should be noted that parameters affecting ^31^P NMR, such as temperature and pH as well as sample preparation, will affect the final quantitative results of ^31^P NMR. For example, PC and PI overlapped at 10 °C and pH 7.6, and PE, SM, and PS were poorly resolved at 50 °C for PL standard mixtures [41]. Furthermore, due to the complexity of HM composition, the chemical shifts of PLs may not be stable, resulting in difficulty in identifying each PL peak.

## 4. Materials and Methods

### 4.1. Chemicals and Reagents

Anhydrous sodium sulfate was purchased from Junsei Chemical Co., Ltd. (Tokyo, Japan). The following HPLC grade solvents, such as methanol, chloroform, isopropanol, hexane, water, which were all purchased from Fisher Scientific Korea Ltd. (Seoul, Korea). Diethyl ether was obtained from Daejung Chemicals & Metals Co., Ltd. (Siheung, Korea). Triundecanoin (C11:0, the internal standard for fat content analysis) was obtained from Nu-Chek Prep, Inc. (Elysian, MN, USA). Ethylenediaminetetraacetic acid (EDTA), triphenyl phosphate (TPP ≥ 99%), chloroform-d (CDCl_3_), and the analytical standards including L-α-phosphatidylethanolamine (P7943-25MG), L-α-phosphatidylcholine (P3556-25MG), L-α-lysophosphatidylcholine (62962-50MG), L-α-phosphatidyl-L-serine (P0474-25MG), and sphingomyelin (85615-50MG) were purchased from Sigma-Aldrich Korea (Seoul, Korea).

### 4.2. Human Milk and Infant Formula Samples

Thirty-four HM samples collected from Maeil Dairies Co. were classified into three groups [i.e., A (*n* = 11), B (*n* = 11), and C (*n* = 12)] according to their fat content. To obtain their fat content, 1 g of HM was subjected to crude fat extraction and methylation before analyzing by an Agilent 6890 Gas Chromatograph (Agilent Technologies Inc., Santa Clara, CA, USA) fitted with a flame ionization detector. A SPTM-2560 capillary column (biscyanopropyl polysiloxane, 100 m × 0.25 mm, 0.25 μm film thickness, Supelco, Bellefonte, PA, USA) was used. The internal standard (triundecanoin, C11:0, 5 mg/mL in iso-octane) was used to calculate the fat content (g/100 g). The infant formula (test product, powder form, based on bovine milk) was provided by Maeil Asia Human Milk R&D Center. The descriptions of the HM samples and mother’s characteristic were as follows: lactation stages, 42–264 days; gestational ages, ≥35 weeks; sexes of the infants, 15 males and 19 females; time of collection, morning and afternoon; single birth; mother’s age, 23–41 years old; mother’s education, high school or above. The donors received the manual breast pump sent from Maeil Asia Human Milk R&D Center, and used it to take samples in the morning and afternoon, then they were frozen immediately. Finally, the HM samples were sent to Maeil Asia Human Milk R&D Center in a box with ice. The criteria for excluding donors included taking any supplements and non-Korean natives. The study design was authorized by the Institutional Review Boards at Maeil Dairies Co., Ltd. (0627-201306-HRBR-001-02, Pyeongtaek-si, Korea) and Chungnam National University (201808-BR-125-01, Daejeon, Korea).

### 4.3. Particle Size Analysis in Human Milk

A laser diffraction analyzer (Mastersizer S, Malvern Instrument Ltd., Worcestershire, UK) was used to measure the milk droplet size. The size of milk fat globule was reported as the surface-weighted mean diameter (d_32_ = Σnid_i_^3^/Σnid_i_^2^) and volume-weighted mean diameter (d_43_ = Σnid_i_^4^/Σnid_i_^3^), where n_i_ is the number of particles with diameter (d_i_). Each milk sample was analyzed in triplicate. Additionally, an optical microscope (CX21, Olympus Optical Co. Ltd., Tokyo, Japan) with ×1000 magnification was used to observe the fat droplets in HM.

### 4.4. Fat Extraction

The extraction of crude fat in HM and infant formula were carried out according to the Folch method [42] with slight modifications. Briefly, 0.5 g of infant formula (test product, powder form) or 3 g of HM sample was thoroughly dissolved in 6 mL of distilled water, and 24 mL of chloroform/methanol (2/1, *v/v*) was added, vortexed for 2 min and then centrifuged for 10 min at 3000 rpm. After centrifugation, the lower chloroform layer was transferred to a new 25 mL vial through an anhydrous sodium sulfate column and the process was repeated adding 12 mL of chloroform and 1 mL of methanol to the upper phase. The two chloroform phases were pooled and flushed with nitrogen at room temperature, and stored at −20 °C until analysis. Each sample was extracted in duplicate.

### 4.5. Phospholipids Analysis

The PLs were separated and quantified [20] with slight modifications. A normal-phase HPLC (Yonglin SP930D) on a YMC-Pack PVA-Sil column (S-5 μm, 250 × 4.6 mml. D. YMC CO., LT) was used with an evaporative light scattering detector (ELSD, ZAM3000, Schambeck SFD GmbH, Bad Honnef, Germany). The solvent system consists of solvent A (isopropanol), solvent B (hexane), and solvent C (water). The gradient elution started at 58% solvent A and 42% solvent B for 4 min, then changed to 58% solvent A, 40% solvent B, and 2% solvent C in 7 min, and finally, changed to 52% solvent A, 40% solvent B, and 8% solvent C and held for 13 min. The evaporation temperature was set at 65 °C. Using nitrogen as the nebulizing gas, the flow rate was 2.0 L/min. Before analysis, 100 μL of chloroform/methanol (2/1, *v/v*) was used to dissolve the crude fat of each sample. Twenty microliters were injected, and the total analysis time was 24 min. All analyses were performed at least in duplication.

In order to quantify PLs in HM, the calibration curves of each species of PLs were obtained by applying the power model [19]. Each standard was freshly prepared and five or six concentrations (2, 1, 0.5, 0.25, 0.1, 0.05 mg/mL) were used to obtain the calibration curve. Each calibration range was as follows: PE, Y = 3280.2X^2.2748^ (R^2^ = 1); PC, Y = 7527.6X^1.2125^ (R^2^ = 1); SM, Y = 8824.1X^1.0862^ (R^2^ = 0.9999); PS + LPC, Y = 2373.5X^1.5694^ (R^2^ = 0.9996).

### 4.6. Phospholipids Analysis by ^31^P NMR

HM fat was applied to solid-phase extraction (SPE, Supelclean^TM^ LC-Si SPE Tubes 6mL, 1 g) for increasing the PL concentration before ^31^P NMR analysis [43]. Briefly, 6 mL of hexane was used to pretreat the SPE column and crude fat (approximately 0.8 g) from HM in chloroform/methanol (2/1, *v/v*) was loaded. Afterwards, 20 mL of hexane/diethyl ether (1/1, *v/v*) was applied, and 20 mL of methanol was used to elute PLs. The recovered PLs were evaporated to dryness under nitrogen for ^31^P NMR analysis.

^31^P NMR analysis of PLs in each fat from HM, infant formula, and SPE was performed [10] with slight modifications. Briefly, each 1 mL of TPP (2.8 mg dissolved in 20 mL CDCl_3_), methanol, and EDTA-Na + solution (0.2 mol/L, pH = 7.0) were added in sequence to a 25 mL of vial containing about 0.5 g of each fat. The mixtures were vortexed for 2 min and centrifuged at 3000 rpm for 10 min at room temperature. Finally, the low-phase-(CDCl_3_)-containing PLs were transferred to the NMR tube through an anhydrous sodium sulfate column.

^31^P NMR spectra were obtained from a Bruker Avance III-600 spectrometer (Bruker BioSpin Corp., Billerica, MA, USA) with a Bruker Magnet (Bruker BioSpin Corp., Billerica, MA, USA) operating at 242 MHz. When analyzing samples, the inverse gating decoupling is used to suppress the nuclear Overhauser effect. ^31^P NMR conditions for PL analysis were as follows: probe temperature, 25 °C; excitation pulse, 30°; the number of data points, 64 K; relaxation delay, 2 s; pulse width, 11.05 μs; acquisition time, 0.34 s; and number of scans, 256. The chemical shifts relative to the TPP internal standard (δ = −17.8) were used to identify the PL species by comparing the PL standard (PE, PC, SM, PS, and LPC) spectra with the sample spectra. SpecMan software (Advanced Chemistry Development Inc., Toronto, ON, Canada) was used to perform the data analysis.

### 4.7. Statistical Analysis

All the data are represented as the mean ± SD. The one-way ANOVA was used to perform the data analysis, followed by Duncan multiple-range tests used to identify significant differences at *p* < 0.05. The correlations between fat content and the contents of total PLs, and between PL species in different groups were determined using the Pearman’s rank correlation coefficient (*r*) and fit using the linearity. All the analyses in this study were carried out using the Statistical Package for the Social Sciences (SPSS Inc., Chicago, IL, USA).

## 5. Conclusions

In this study, the content of total PLs (sum of SM, PC, PE, and PS + LPC) and each PL species in Korean HM was quantified, wherein SM was the most abundant PL (5.11 ± 1.42 mg/100 g), followed by PC (2.26 ± 0.55 mg/100 g) and PE (2.00 ± 0.84 mg/100 g). When comparing the classified groups (A, B, and C), the content of PL (mg/100 g HM) was significantly higher in the C group with the highest fat content (*p* < 0.05), suggesting that there is a positive relationship between fat content and PL content in HM. However, the normalized PL content (mg/g fat) was significantly higher in group A with low fat content (*p* < 0.05), and the fat droplet size in group C was larger than that in group A without a statistical difference (*p* > 0.05). Inferring from the above results, while fat content of HM is greatly influenced by the number of fat droplets, it is also affected by fat droplet size to an extent that it cannot be ignored. In addition, the correlations (*r*) between PL species were explored, and SM and PC exhibited a correlation in all groups, and the correlation extent between PE and either PC or SM was in the order of C > B > A, indicating that the correlations between PL species are related to the fat content of HM. It is believed that PC and PE are biosynthesized from choline and ethanolamine, which should be largely provided by the diet, and they participate in SM synthesis due to interconversion along their own biosynthetic pathways. Our observation that the composition and content of PL species in HM varied according to its fat content implicates the importance of maternal diet and the need to consume choline and ethanolamine in the diet. Therefore, the diet affects the fat content of HM, even though genetic differences in the individuals should also be considered. In addition, the feasibility of ^31^P NMR quantification of the contents of PLs in HM was explored. The results showed that HPLC-ELSD is considered as the recommended quantitative method instead of ^31^P NMR for samples containing low concentrations of PLs (% of fat), as is the case with HM. Otherwise, it is recommended to analyze PL contents by ^31^P NMR after increasing the relative contents of PLs in HM through SPE treatment.

## Figures and Tables

**Table 1:** Phospholipid (PL) concentrations in human milk (HM) from the lactating mothers in Korea.

	Total Mature Milk ^1^					
PL species ^2^	mg/100 g HM	Range	mg/g fat	Range	% of total PLs ^3^	Range
PE	2.00 ± 0.84	0.68–4.21	0.52 ± 0.21	0.24–1.16	18.89 ± 3.93	9.21–24.42
PC	2.26 ± 0.55	1.13–3.14	0.64 ± 0.30	0.30–1.46	22.41 ± 3.83	17.38–32.04
SM	5.11 ± 1.42	2.35–8.32	1.35 ± 0.49	0.71–2.59	49.64 ± 3.90	40.64–56.15
PS + LPC	1.01 ± 0.62	0.20–2.19	0.24 ± 0.09	0.07–0.47	9.06 ± 3.75	3.34–17.78
Total PLs	10.39 ± 3.11	4.89–17.85	2.74 ± 0.94	1.39–5.45	-	-

^1^ One sample in group B was omitted due to insufficient amount for analysis. ^2^ PE, phosphatidylethanolamine; PC, phosphatidylcholine; SM, sphingomyelin; PS, phosphatidylserine; LPC, lysophosphatidylcholine. ^3^ Total PLs, sum of PE, PC, SM, and PS + LPC. Data represented as mean ± SD.

## Supplementary Materials

Table S1: Phospholipids (PL) concentrations in human milk (HM) and infant formula obtained from HPLC-ELSD and ^31^P NMR, Figure S1: HPLC-ELSD chromatograms and ^31^P NMR spectra. Standard mixtures, a and a’; fat from human milk, b and b’; fat from infant formula, c and c’. 1, phosphatidylethanolamine (PE); 2, phosphatidylcholine (PC); 3, sphingomyelin (SM); 4, lysophosphatidylcholine (LPC); 5, phosphatidylserine (PS); 6, PS + LPC. The variability of ^31^P NMR chemical shifts were as follows: PC (−0.76–−0.84 ppm); LPC (−0.12–−0.35 ppm); SM (−0.02–0.04 ppm); PS (−0.21–0.05 ppm); PE (−0.13–0.18 ppm).

## Sample Availability

Not available.
