# Supplementary material for: Correlations of Fat Content in Human Milk with Fat Droplet Size and Phospholipid Species
Source: Molecules. 2021 Mar 13;26(6):1596. doi: 10.3390/molecules26061596 (PMC8000790; doi:10.3390/molecules26061596)
Supplement: Supplementary file 1 [file molecules-26-01596-s001.pdf]

Table S1. Phospholipids (PL) concentrations in human milk (HM) and infant formula obtained from HPLC-ELSD and ^{31}P NMR.

PL species [1]	PL concentrations (mg/100 g)			PL concentrations (% of fat)		
	**HPLC-ELSD**	**^{31}P NMR**	**RSD (%)**	**HPLC-ELSD**	**^{31}P NMR**	**RSD (%)**
Human milk						
PE	2.74 ± 0.75	ND [2]	-	0.04 ± 0.11	ND	-
PC	2.52 ± 0.53	4.59 ± 0.22	41.23	0.04 ± 0.01	0.08 ± 0.00	44.08
SM	5.73 ± 1.29	8.31 ± 0.69	25.97	0.09 ± 0.02	0.14 ± 0.01	29.70
PS + LPC	1.45 ± 0.53	ND	-	0.02 ± 0.01	ND	-
Total	12.44 ± 1.11	12.90 ± 0.91	2.58	0.19 ± 0.02	0.21 ± 0.02	8.43
Infant formula (test product, powder form)						
PE	83.80 ± 3.45	73.54 ± 9.97	9.23	0.35 ± 0.01	0.33 ± 0.05	2.81
PC	403.99 ± 6.69	372.76 ± 18.69	5.69	1.67 ± 0.03	1.70 ± 0.11	1.29
SM	72.28 ± 0.29	56.98 ± 12.38	16.74	0.30 ± 0.00	0.26 ± 0.06	11.45
PS + LPC	38.77 ± 3.55	44.13 ± 1.47	9.15	0.16 ± 0.01	0.20 ± 0.00	15.97
Total	598.84 ± 6.51	547.41 ± 5.12	6.35	2.47 ± 0.03	2.48 ± 0.01	0.37

[1] PE, phosphatidylethanolamine; PC, phosphatidylcholine; SM, sphingomyelin; PS, phosphatidylserine; LPC, lysophosphatidylcholine. [2] ND, not detected.

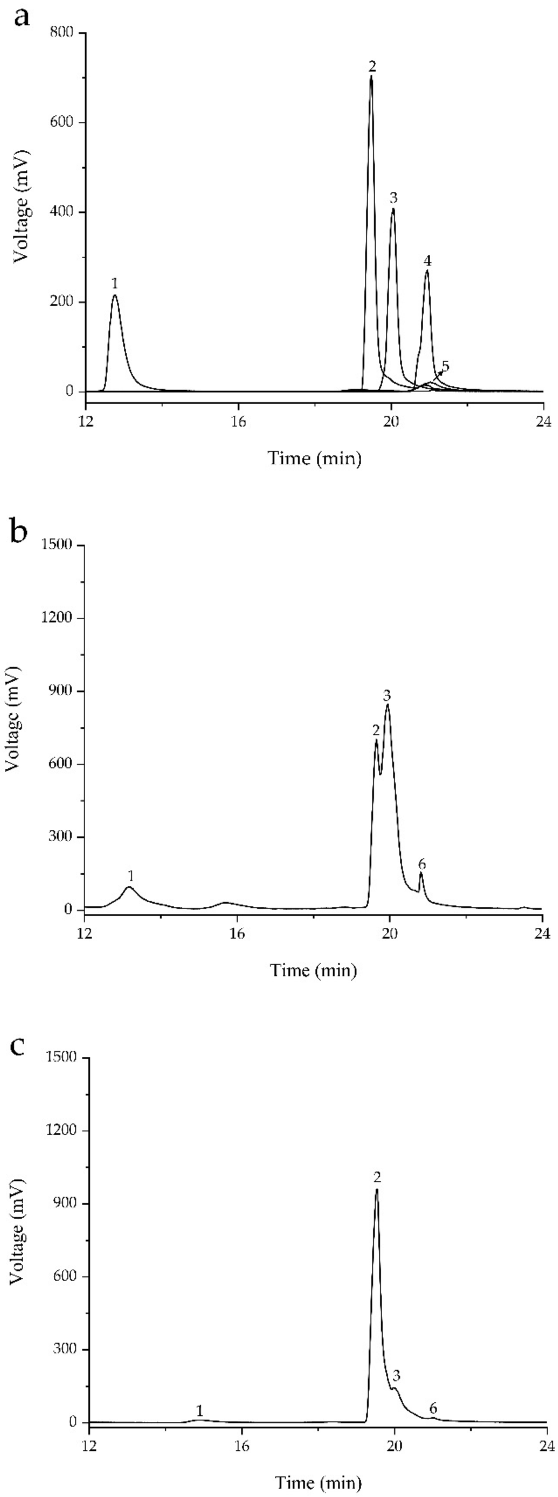
a
Voltage (mV)
800
600
400
200
0
1
2
3
4
5
12
16
20
24
Time (min)
b
Voltage (mV)
1500
1200
900
600
300
0
1
2
3
6
12
16
20
24
Time (min)
c
Voltage (mV)
1500
1200
900
600
300
0
1
2
3
6
12
16
20
24
Time (min)

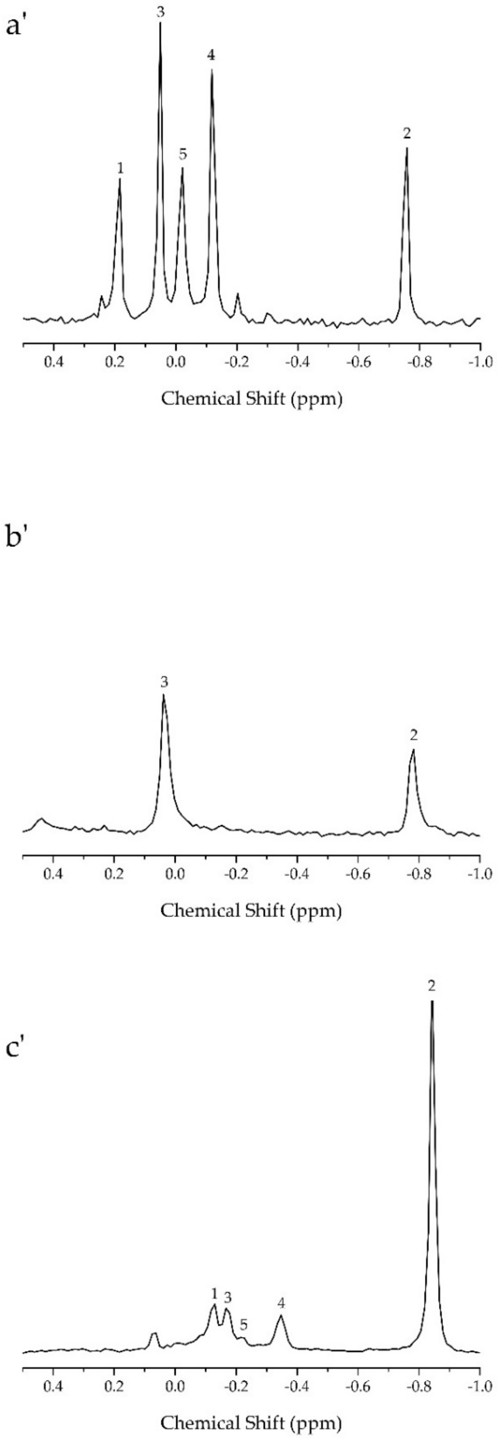
a'
3
4
2
5
1
0.4 0.2 0.0 -0.2 -0.4 -0.6 -0.8 -1.0
Chemical Shift (ppm)
b'
3
2
0.4 0.2 0.0 -0.2 -0.4 -0.6 -0.8 -1.0
Chemical Shift (ppm)
c'
2
1 3
5
4
0.4 0.2 0.0 -0.2 -0.4 -0.6 -0.8 -1.0
Chemical Shift (ppm)

Figure S1. HPLC-ELSD chromatograms and ^{31}P NMR spectra. Standard mixtures, a and a'; fat from human milk, b and b'; fat from infant formula, c and c'. 1, phosphatidylethanolamine (PE); 2, phosphatidylcholine (PC); 3, sphingomyelin (SM); 4, lysophosphatidylcholine (LPC); 5, phosphatidylserine (PS); 6, PS + LPC. The variability of ^{31}P NMR chemical shifts were as follows: PC (-0.84– -0.76 ppm); LPC (-0.35– -0.12 ppm); SM (-0.02–0.04 ppm); PS (-0.21–0.05 ppm); PE (-0.13–0.18 ppm).
